# University of North Carolina/Emory Center for Innovative Technology (iTech) for Addressing the HIV Epidemic Among Adolescents and Young Adults in the United States: Protocol and Rationale for Center Development

**DOI:** 10.2196/10365

**Published:** 2018-08-03

**Authors:** Lisa B Hightow-Weidman, Kathryn Muessig, Eli Rosenberg, Travis Sanchez, Sara LeGrand, Laura Gravens, Patrick S Sullivan

**Affiliations:** ^1^ Behavior and Technology Lab, Institute for Global Health and Infectious Diseases University of North Carolina at Chapel Hill Chapel Hill, NC United States; ^2^ Department of Health Behavior University of North Carolina at Chapel Hill Chapel Hill, NC United States; ^3^ Department of Epidemiology and Biostatistics State University of New York at Albany Albany, NY United States; ^4^ Department of Epidemiology Rollins School of Public Health Emory University Atlanta, GA United States; ^5^ Duke Global Health Institute Duke University Durham, NC United States

**Keywords:** adolescent, HIV, care continuum, technology, mobile app

## Abstract

**Background:**

Over a fifth of all new HIV infections in the United States occur among persons aged 13 24 years, with most of these diagnoses occurring among gay and bisexual males (81%). While the epidemic of HIV in the United States has leveled off for many age groups, the annual number of new HIV diagnoses among young men who have sex with men (YMSM; 13-24 years old) remains high. Traditional approaches to continuum improvement for youth have been insufficient, and targeted interventions are urgently needed for young people at risk for or infected with HIV. Interventions delivered through mobile health technology represent a promising approach for improving outcomes in this population. Mobile phones have nearly reached saturation among youth, making mobile technology a particularly promising tool for reaching this population.

**Objective:**

The University of North Carolina/Emory Center for Innovative Technology (iTech) is a National Institutes of Health cooperative agreement as part of the Adolescent Medicine Trials Network for HIV/AIDS Interventions. iTech aims to impact the HIV epidemic by conducting innovative, interdisciplinary research on technology-based interventions across the HIV prevention and care continuum for adolescents and young adults in the United States, particularly YMSM, by providing the following: (1) evaluation of novel approaches to identifying youth with undiagnosed HIV infections; (2) evaluation of multilevel, combination prevention approaches, particularly relevant to gender- and sexual-minority youth facing co-occurring health risks; (3) evaluation of uptake of and adherence to biomedical prevention modalities; and 4) evaluation of interventions designed to promote or optimize engagement in care and antiretroviral therapy adherence in HIV-positive youth, to optimize viral load suppression.

**Methods:**

iTech brings together multidisciplinary experts in the fields of adolescent HIV treatment and prevention, development and evaluation of technology-based interventions, HIV surveillance and epidemiology, and intervention design and evaluation. This initiative will support 8 efficacy trials and 2 exploratory projects, each led by 2 principal investigators. Taken together, the studies address all of the key steps of the HIV prevention and care continuum for youth in the United States. Each proposal uses technology in a scientifically rigorous and innovative way to access, engage, and impact at-risk or infected youth. Nine iTech subject recruitment venues are spread across 8 US cities. Three cores (management, analytic, and technology) support all iTech activities and form the research network’s infrastructure, facilitating all aspects of study implementation and evaluation.

**Results:**

Formative work has already begun on many of the above-mentioned iTech trials. We expect the first randomized controlled trials to begin in mid-2018. Additional details can be found in the individual intervention protocol papers in this issue.

**Conclusions:**

Through its comprehensive research portfolio, iTech aims to effectively advance HIV prevention and care for youth through technology-based, youth-relevant interventions that maximize adaptability and sustainability.

**Registered Report Identifier:**

RR1-10.2196/10365

## Introduction

Over a fifth (22%) of all new HIV infections in the United States occur among persons aged 13‑24 years, with most of these diagnoses occurring among gay and bisexual males (81%) [1]. While the epidemic of HIV in the United States has leveled off for many age groups, the annual number of new HIV diagnoses among young men who have sex with men (YMSM; 13-24 years old) remains high. Though it fell by 18% between 2008 and 2014, in 2015, YMSM accounted for 92% of new infections among people aged 13‑24 years and 27% of all new infections among MSM [2].

Advances in HIV prevention tools can effectively reduce new HIV infections among youth. Antiretroviral therapy (ART) is a powerful tool and can be used among HIV‑negative youth to reduce susceptibility to infection (pre-exposure prophylaxis, PrEP) or among youth living with HIV infection (in the form of treatment as prevention) to reduce infectiousness [3-6]. The effectiveness of ART for reducing HIV transmission requires successes at multiple steps of the HIV prevention and care continuum (HIV testing, PrEP or ART treatment initiation, and treatment adherence), which may prove challenging for youth due to individual, structural, and societal barriers [7-10]. Comprehensive, evidence-based behavioral, psychosocial, and structural interventions are needed to optimize PrEP and treatment as prevention among youth.

HIV testing is the critical first component to facilitate entry into HIV prevention and care. Individuals who are aware of their infection can begin treatment, thus reducing the likelihood of onward transmission and improving clinical outcomes [11]. Those individuals testing negative can engage in interventions to prevent HIV acquisition, such as behavioral counseling and PrEP initiation. Due to the high HIV incidence among YMSM, routine HIV testing is particularly important and represents an ongoing prevention activity that requires strategies for continued engagement [12]. Access to and uptake of HIV testing is suboptimal, even among YMSM who report behaviors that place them at high risk for infection [13,14]. Data from the 2015 Youth Risk Behavior Surveillance System, which collects data from high school students (9th-12th grade), found that among sexually debuted YMSM, only 21% had ever been tested for HIV [1]. Further, at the end of 2013, an estimated 60,900 youth were living with HIV in the United States. Of these, 51% (31,300/60,900) were living with undiagnosed HIV, the highest rate of undiagnosed HIV in any age group [1].

Although PrEP has demonstrated high efficacy in clinical studies, uptake has been low among YMSM, especially YMSM of color [8,15,16]. There have been a number of challenges to increasing PrEP uptake in the United States, including low awareness of PrEP among youth and providers, only recent FDA approval of PrEP for those aged <18 years, concerns about potential side effects or safety, low risk perception, and PrEP stigma [17-19]. Efficacy of PrEP is highly correlated with adherence, and evidence suggests there may be worse adherence rates among younger populations and racial and ethnic minority populations [20-23].

For youth diagnosed with HIV, engagement in care and treatment with ART to achieve viral suppression are critical components for reaching the goals of improved individual health and prevention of onward transmission. In the United States, only approximately 44% of young people aged 13‑24 years diagnosed with HIV have achieved viral suppression [1]. One recent study of 1548 youths [24], conducted within the Adolescent Trials Network for HIV/AIDS Interventions (ATN), showed that only 7% of diagnosed adolescents and young adults achieved undetectable viral loads. Among participants with baseline biomedical data (N=733), 81.0% (594/733) were male, 72.0% (528/733) were black, and 70.0% (513/733) were gay or bisexual. This was substantially lower than the estimated 50% of persons achieving viral suppression for all age groups combined [24,25].

Thus, traditional approaches to continuum improvement for youth have been insufficient. Targeted interventions are urgently needed to improve the knowledge of undiagnosed HIV infection, access to and retention in prevention and care, medication adherence, and long-term viral load outcomes among youth at risk for or infected with HIV. Interventions delivered through mobile health (mHealth) technology represent a promising approach for improving outcomes among youth at risk for or infected with HIV. Mobile phones have nearly reached saturation among youth, making mobile technology a particularly promising tool to reach this population. As of 2015, 78% of those aged 18-29 years in the United States own a desktop or laptop computer and 98% report having a mobile phone of some kind, and 86% of these devices are smartphones [26]. Although white teens (91%) are more likely than black or Latino teens to report owning a desktop or laptop computer (~80% each), black teens are more likely to own a mobile phone (85%) and go online through a mobile device (100%) than white teens (71% ownership, 90% access) [27].

Although a number of apps related to HIV and other sexually transmitted infections (STIs) are available via commercial sites (Apple iTunes, Google Play), there are limited data to support the design of these apps or rigorous evaluations of their impact [28,29]. Of note, there are no current mHealth interventions targeting access to, uptake of, and adherence to PrEP. Further, youth face unique barriers to care at multiple levels including individual, social, and structural. Thus, technology (including app-based) interventions for youth must be speciﬁcally tailored based on their developmental stage and should include factors beyond individual-level behaviors and barriers to care. Additionally, once developed, these interventions must be rigorously evaluated and include the estimation of both overall cost and cost-effectiveness (compared with standard of care).

To effectively advance HIV prevention for youth, technology-based, youth-relevant interventions that maximize adaptability and sustainability need to be developed, heeding lessons learned from successes and failures of in-person interventions. A more efficient and potentially more scalable process for developing and testing these interventions would be to collate interventions that share common principles, design elements, data collection, and evaluation approaches. Moving forward, it is imperative that we build upon and enhance successful platforms to develop sustainable technology-based solutions that represent the highest standards of usability, affordability, accessibility, and large-scale dissemination.

The University of North Carolina (UNC)/Emory Center for Innovative Technology (iTech) is a National Institutes of Health cooperative agreement as part of the ATN (see Eunice Kennedy Shriver National Institute of Child Health and Human Development ATN overview, also submitted for this special issue). iTech aims to impact the HIV epidemic by conducting innovative, interdisciplinary research on technology-based interventions across the HIV prevention and care continuum for adolescents and young adults in the United States. The overall goals of iTech seek to decrease the impact of HIV on the lives of adolescents and young adults in the United States, particularly YMSM, by providing the following: (1) evaluation of novel approaches to identifying youth with undiagnosed HIV infection; 2) evaluation of multilevel, combination prevention approaches, particularly relevant to gender and sexual-minority youth facing co-occurring health risks (eg, substance use, mental illness, homelessness; 3) evaluation of uptake of and adherence to biomedical prevention modalities; and 4) evaluation of interventions designed to promote or optimize engagement in care and ART adherence in HIV-positive youth, to optimize viral load suppression.

## Methods

### iTech Structure

iTech will support 8 efficacy trials of interventions and 2 exploratory projects, each of which will be led by 2 principal investigators (PIs). Taken together, the studies address all of the key steps of the HIV prevention and care continuum for youth in the United States. Each proposal uses technology in a scientifically rigorous and innovative way to access, engage, and impact at-risk or infected youth. Nine iTech subject recruitment venues (SRVs) are spread across 8 US cities (Boston, MA; Philadelphia, PA; Chicago, IL; New York City, NY, 2 sites; Houston, TX; Tampa, FL; Atlanta, GA; and Los Angeles, CA). Three cores (management, analytic, and technology) support all iTech activities and form the research network’s infrastructure that supports all aspects of study implementation and evaluation.

#### iTech Cores

**Figure 1:**
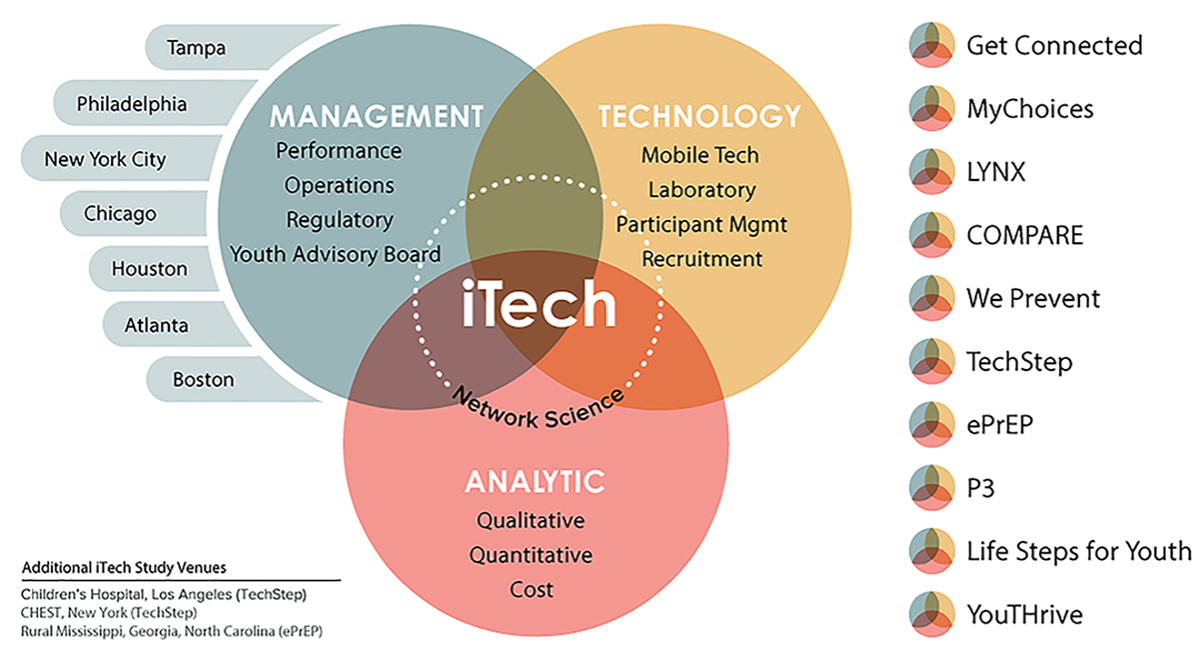
Overall iTech organizational structure.

iTech is composed of three cores (management, analytic, and technology) that function in a coordinated and complementary manner to achieve overall objectives (Figure 1). Authors LBH-W and PSS oversee all activities. A project management plan provides rules for iTech Core governance.

The *Management Core* (MC) provides the organization and structure necessary to maximize the potential of the research projects within iTech. MC provides infrastructure, regulatory, and operational support and ensures communication and collaboration among the research studies within iTech and with the funders. It is responsible for the project management plan and overall and study-specific timelines, ensuring the project remains within cost and scope, and overseeing and monitoring the iTech SRVs.

The *iTech Analytic Core* (AC) provides expertise and data systems for the conduct of formative research, usability testing, pilot studies, randomized controlled trials (RCTs) and economic analyses to support the aims of the iTech and its research protocols. Throughout the development, execution, analysis, and dissemination phases of each iTech study activity, AC provides guidance on implementing cross-site collaborative research, maintaining scientific rigor, and ensuring timely research completion. Activities of AC are supported by data analytic staff at Emory and UNC with skills in 4 primary areas: data management, qualitative data analysis, quantitative data analysis, and costing analyses. Lead statisticians assemble and supervise teams comprising AC data management and analytic staff for each RCT and complete the design and execution of primary analyses for each iTech study. AC also leads efforts to ensure study tools and measures are harmonized wherever possible across iTech studies and the broader ATN to facilitate pooled and large-scale secondary data analyses projects. Network-wide measures encompass 5 domains: demographics or socioeconomic characteristics, sexual behavior and risk, substance use or abuse, HIV-positive cascade, and HIV-negative cascade. In addition, other survey measures specifically for use within iTech were developed: additional demographic or socioeconomic characteristics, technology items (technology use, eHealth literacy, intervention usability, and intervention acceptability), mental health, and social supports. Further details on ATN data harmonization can be found in a companion manuscript in this eCollection (will be cited once available).

Economic analyses are planned for five of iTech’s current studies (COMPARE, P3, Get Connected, ePrEP, and YouTHrive). Cost data will be collected by input type and activity. Standardized tools and harmonized measures will be used across studies wherever possible. The study staff will develop the inventory of inputs for each activity for each intervention (eg, inputs for the initial screening visit and app setup) and standard definitions of each input and unit of measure. Each study site will use a standard data collection instrument that lists all the inputs, the time frame for collection, and the primary source of cost data. We will use activity-based (also called “bottom-up”) costing to assess the cost of the respective interventions. Cost data will be collected by site. The average of the site-specific costs will be used, and the variation in site-specific costs will be examined and reported. Total cost results will be presented by cost per participant using the intervention and cost per percent increase in primary study outcome(s) (eg, HIV testing, PrEP uptake), referent to the control arm.

*The iTech Technology Core* (TC) provides services for technology-related fields, including mobile technologies, Web-based platforms, laboratory monitoring platforms that collect samples through mailed-out testing kits, social media- and Web-based recruitments, and technology-related ethics issues. TC provides a secure, HIPPA (Health Insurance Portability and Accountability Act of 1996)-compliant Web-based system for participant management and retention that is used across all SRVs and studies. TC assists with the measurement of *paradata*, auxiliary data that capture details about the process of interaction with the Web-based intervention, including website and app analytics, and creates an infrastructure to share and disseminate best practices in technology-delivered HIV interventions. To date, paradata have been underexamined and underreported in research among youth at risk for or infected with HIV [30]. Paradata can be used to help understand what components of the intervention led to behavior change and which components are not useful and should not be continued in future iterations of the technology. Finally, TC provides support for scaling and dissemination, including planning for eventual implementation at all stages of protocol development.

#### Commitment to Adolescent Research

Through a developed and centralized management and analytic framework, iTech supports a network of multidisciplinary scientists, researchers, clinicians, and public health professionals who work collaboratively to support iTech’s diverse research portfolio. The network science team is made up of all research study PIs, SRV PIs, plus members of iTech’s scientific leadership cores. This team meets monthly to talk about cross-cutting issues, brainstorm new concepts, review new scientific research, and leverage cross-protocol opportunities. iTech also supports a collaborative training and educational mission that crosses all cores and disciplines. iTech provides a nurturing environment to foster new junior investigators into adolescent HIV or AIDS research and builds collaborations to create new and innovative research in this area. We have deliberately built mentoring relationships into each of the research studies, pairing junior investigators with more experienced investigators.

#### Community Engagement

iTech has purposefully partnered with clinical care sites that have well-established community collaborations, including established youth advisory boards (YABs). We will work closely with each SRV to ensure sustained YAB involvement at each venue and within the larger iTech network. iTech’s leadership will also work closely with YABs and other community stakeholders and advisors to help evaluate tools and programs, ensuring age appropriateness and readability, cultural sensitivity and linguistic appropriateness, gender and sexual orientation sensitivity, and minimal burden of intervention strategies. In addition, iTech has proposed involvement of at least one YAB member from each SRV in monthly Web-based Youth Advisory Council meetings to allow input on local as well as cross-iTech issues.

#### Regulatory and Ethics

All research conducted within iTech will be centrally reviewed and approved by the institutional review board (IRB) of the University of North Carolina at Chapel Hill, acting as the IRB of record in accord with National Institutes of Health policies [31]. Local IRBs for participating institutions and SRVs sign reliance agreements with the UNC IRB and receive regular updates and notices of continuations or changes in protocol, as well as are informed of any adverse or serious adverse events that may occur at their site. A waiver of parental consent or assent is obtained for participants who are 15-17 years old. All studies have (5 studies) or will be (5 studies) registered on ClinicalTrials.gov (Table 1).

#### iTech Projects

**Figure 2:**
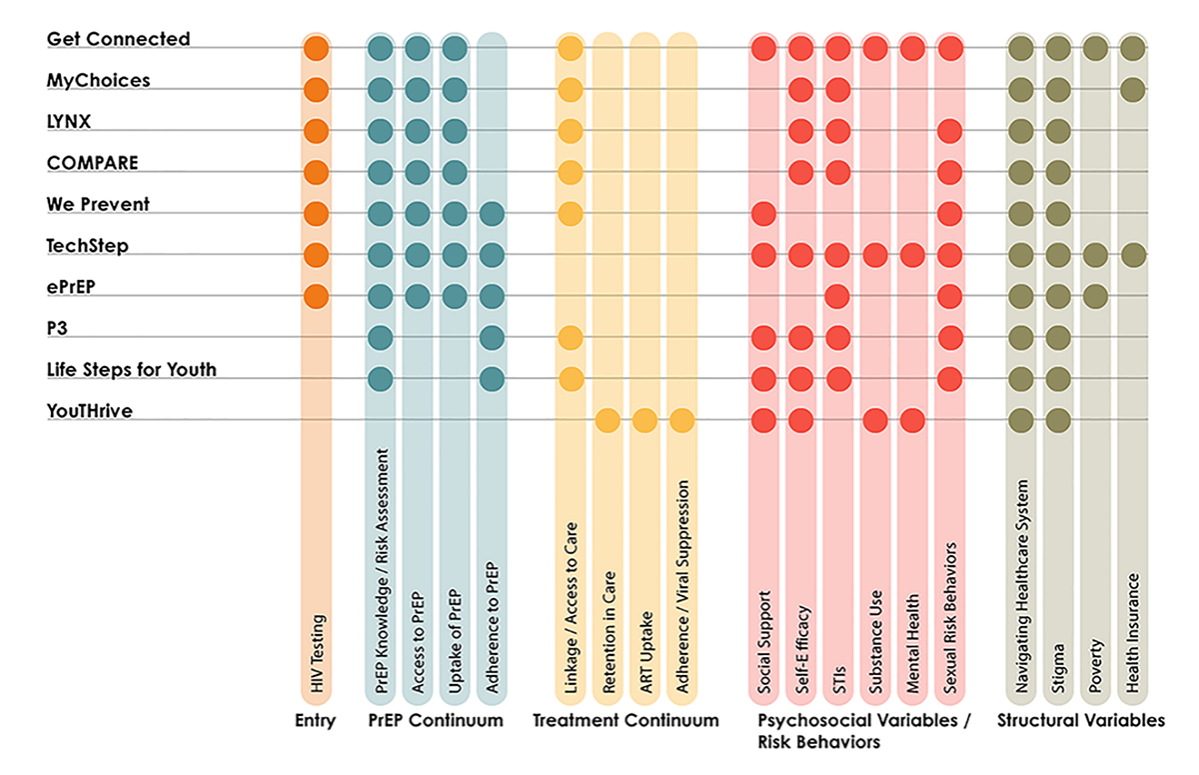
iTech research studies and intended continuum of prevention and care targets. ART: antiretroviral therapy; PrEP: pre-exposure prophylaxis; STI: sexually transmitted infection.

The iTech scientific agenda is translated through 8 efficacy trials and 2 exploratory projects, all designed to address 4 primary objectives. Each study includes qualitative formative research to develop or adapt the intervention and an RCT of the intervention(s) versus standard of care. Many new or substantially adapted interventions are also conducting technical pilots in which the intervention is tested for a short period of time and qualitative exit interview data are used to finalize the intervention for RCT. This standardization of the research process across iTech studies is a key aspect of the network. Below, we outline each of the 4 objectives and briefly describe the interventions currently in place to address these objectives; note that all iTech interventions address multiple components of the care and prevention cascades and cross-cutting issues (Figure 2). Additional details can be found in the individual intervention protocol papers (will be cited once available or under review).

### Evaluation of Novel Approaches for Identifying Youth With Undiagnosed HIV Infection

Despite Centers for Disease Control and Prevention and American Academy of Pediatrics recommendations that high-risk youth receive an HIV test at least annually [32], many sexually active YMSM have either never been tested or have not been tested in the last year. More than half of youth living with HIV (51%) are unaware of their infection [1,14,33,34]. Barriers to testing among youth, including YMSM, include misperception of individual risk, fear of testing positive, concerns about confidentiality, access to healthcare services, and provider reluctance to discuss sexual risk behaviors among adolescent patients and offer routine testing to those at risk [35]. Four iTech studies have a primary focus on HIV testing:

- *LYNX*: LYNX is a novel mobile app designed to increase HIV or STI testing and support PrEP uptake among YMSM. In this study, the investigators will expand their current mobile app, designed primarily to increase HIV or STI testing, to include components to increase the uptake and linkage to PrEP for YMSM, and then evaluate the feasibility and acceptability of this app in a pilot RCT. The key components addressed by the integrated LYNX app will be information, motivation, and behavioral skill needs [36-38] for increasing HIV or STI testing frequency and PrEP uptake.
- *MyChoices*: MyChoices is a youth-optimized version of a mobile app designed to increase HIV or STI testing and support PrEP uptake among YMSM [39]. In this study, the investigators will expand and enhance their current mobile app through theater testing with youth and then evaluate the feasibility and acceptability of this app in a pilot RCT. The key components of MyChoices address constructs of social cognitive theory, including self‑regulation, self-efficacy, and environmental influences [40].
- *COMPARE*: If either app described above (or both) is shown to be feasible and acceptable, the app(s) will be tested in this follow-on research study to evaluate for efficacy. If both are deemed feasible and acceptable, YMSM in this 3-year study will be randomized to receive either MyChoices or LYNX or standard of care information about HIV testing and PrEP.
- *We Prevent*: This project aims to develop and test a relationship skills-focused HIV prevention intervention for YMSM and their partners. The intervention consists of two telemedicine sessions: the first focuses on relationship skills, and the second consists of HIV testing and counseling for couples and prevention planning [41]. Both sessions are attended by both members of the dyad.

**Table 1:** iTech studies’ registration status.

Study name	ClinicalTrials.gov identifier or anticipated date of registration
Get Connected	NCT03132415
LYNX	NCT03177512
MyChoices	NCT03179319
P3 (Prepared, Protected, emPowered)	NCT03320512
YouthThrive	NCT03149757
LifeSteps for PrEP for Youth	Mid-2018
We Prevent	Mid-2018
ePrEP	Mid-2018
TechStep	Mid-2018
COMPARE	Late 2018

### Evaluation of Multilevel, Combination Prevention Approaches

Structural factors, such as lack of access to or prior negative experiences with prevention and care services may impede HIV or STI testing and PrEP uptake among YMSM and transgender youth, particularly among youth with co-occurring health risks and comorbidities (eg, substance use, mental illness, homelessness) [42-44]. It is critical to provide developmentally and culturally appropriate services and tailored short message service (SMS) text messaging for YMSM and transgender youth, to increase their engagement in risk reduction activities, routine uptake of HIV or STI testing, and awareness of PrEP. Two iTech studies primarily focus on combination prevention approaches that address structural factors:

- *Get Connected*: Get Connected! is a motivationally based, brief Web-based intervention that employs individual and system-level tailoring technology to reduce barriers to linkage into competent prevention care (eg, HIV or STI testing, PrEP) for YMSM [45,46]. YMSM receive personalized and theory-driven content regarding HIV or STI prevention, and structural factors are addressed through linkage to prevention and care services known to serve YMSM populations competently.
- *TechStep*: The rates of HIV infection among transgender youth (including transfeminine, transmasculine, and gender-nonconforming youth) are extremely high, particularly among transwomen [47,48] and transmen who have sex with men [49]. Furthermore, engagement in routine health care has been problematic due to structural barriers (eg, housing instability, unemployment or underemployment, limited educational attainment), provider attitudes, and perceived or actual experiences of stigma and discrimination [50]. TechStep is a two‑condition, technology-based RCT, with a stepped care approach, among high-risk HIV-negative transgender youth for reducing sexual risk behaviors and increasing PrEP uptake. The stepped care approach includes increasing intervention intensity from SMS text messaging or a Web app to e-coaching.

### Evaluation of Uptake of and Adherence to Biomedical Prevention Modalities

PrEP provides a strong preventive benefit to youth at risk for HIV infection and there is overwhelming evidence supporting its efficacy [20,21,51-53]. Although oral PrEP adherence is highly correlated with its efficacy in clinical trials, adherence rates are variable [52,54-56]. In real-world practice settings, PrEP adherence may even be lower, particularly among youth [21,23]. As such, interventions are needed to improve and sustain adherence to PrEP, thereby maximizing its preventive benefits in at risk populations. Three iTech interventions primarily address PrEP uptake and adherence:

- *LifeSteps for PrEP for Youth (LSPY)*: LifeSteps is an evidence-based HIV medication adherence intervention for HIV infected individuals, which was developed by Safren et al [57-59] and has been adapted for diverse populations [60-62], including adolescents [63]. It consists of four, weekly, nurse-delivered sessions with weekly SMS. In this study, investigators will tailor LSPY to meet the unique needs of YMSM and then conduct a pilot two-arm RCT of the modified version of LSPY to test feasibility, acceptability, and preliminary efficacy.
- *P3 (Prepared, Protected, emPowered)*: P3 is an interactive mobile phone app for HIV-uninfected YMSM. The app utilizes social networking and game-based mechanics, as well as a comprehensive understanding of what constitutes “best practices” in app development to improve PrEP adherence and retention in preventive care. An enhanced arm (P3+) will deliver in-app adherence counseling based on the integrated Next Step Counseling model [64,65]. A three-arm RCT will be conducted.
- *ePrEP*: Rural and periurban areas across the Southeast do not have extensive access to PrEP providers. A tailored approach for rural YMSM, addressing known structural barriers of transportation, access to providers, and privacy, is likely to yield high levels of PrEP initiation and persistence in care. The study will finalize the development of a unified rural telemedicine system with a standalone mobile phone app interface and then conduct an RCT of the ePrEP intervention to determine if there is higher PrEP adherence compared with a control condition, which gives access to a Web-based PrEP locator.

### Evaluation of Interventions Designed to Promote or Optimize Engagement in Care, ART Adherence, and Viral Load Suppression in HIV-Positive Youth

Poor adherence among youth is multifactorial and includes medical (eg, side effects, dissatisfaction with medical team), logistical (eg, forgetting, inconvenience), and psychological (eg, depression, lack of support, perceived stigma) barriers [66,67]. Sufficient and sustained ART adherence reduces excess morbidity and mortality among people living with HIV [68] and lowers the probability of forward transmission to sexual partners [69]. Advancing targeted and innovative ART adherence interventions for youth with HIV is an urgent priority; one iTech intervention primarily targets ART adherence:

- *YouTHrive*: YouTHrive (pronounced “youth thrive”) is a Web app intervention to improve ART adherence among youth living with HIV that has the following components: (1) enhanced peer-to-peer interaction, (2) engagement SMS text messages, (3) mood and ART adherence self-monitoring, (4) goal setting, and (5) tailored ART and HIV informational content. Gamification techniques (eg, leveling) are used to promote sustained engagement.

## Results

Formative work has already begun on a number of the iTech trials detailed above. We expect the first RCTs to begin in mid-2018. iTech is anticipating preliminary findings from the first randomized control trials (LYNX and MyChoices) to be presented by early 2019. Additional preliminary findings from TechStep and P3 are expected mid to late 2020, with YouTHrive, Get Connected, and ePrEP presenting preliminary RCT findings by late 2021. We expect additional findings as we add new interventions focused within each of our four iTech objectives.

## Discussion

iTech brings together multidisciplinary experts in the fields of adolescent HIV treatment and prevention, development and evaluation of technology-based interventions, HIV surveillance and epidemiology, and intervention design and evaluation. Fostered by an MC that prioritizes communication and collaboration, this robust team will work collaboratively to respond to emerging issues and promote continued advancements in the field.

Technology-delivered interventions are well-suited for youth given their modality, the common use of technology in the population, the platform’s suitability to deliver tailored content specific to each user’s HIV or AIDS risk behaviors and context, and the platform’s unique capability to diffuse HIV or AIDS prevention programs to large numbers of youth residing in numerous geographic locations. Youth, including YMSM, are receptive to internet- and mobile phone-delivered interventions [29,70,71].

Using the internet to recruit, engage, and retain youth in interventions is not only possible but also necessary. Youth may lack the social capital or resources to access in-person interventions, may be dependent on adults for money or transportation, or may need permission to attend programs. These barriers are particularly problematic for YMSM who, due to anticipated or actual stigma, are unable or unwilling to talk to adults about their same-sex attractions, behavior, sexual identity, and need to receive prevention and care services [72-75]. Traditional face-to-face formats thus create difficulties for youth when they cannot easily access these interventions in their communities, are not able to attend the sessions when they are offered, and cannot choose to engage with the interventions’ content when most convenient. In addition, in a world where so much content is available online, there is an appeal (and perhaps even an expectation) to interventions that can be accessed online, in the privacy of one’s home, viewed alone or with friends, and when best suits the young person. However, while in-person interventions may be costlier, some youth may benefit from face-to-face interactions with culturally responsive providers. Thus, costing analyses are critically important with regard to technology-based interventions that are resource intensive to develop but have a great potential to be scalable and cost-effective, with high public health impact [76]. We have integrated sophisticated methodologies to translate findings into HIV prevention recommendations for youth in the United States.

## Abbreviations

AC: Analytic Core
ART: antiretroviral therapy
ATN: Adolescent Medicine Trials Network for HIV/AIDS Interventions
HIPPA: Health Insurance Portability and Accountability Act of 1996
IRB: institutional review board
iTech: University of North Carolina/Emory Center for Innovative Technology
LSPY: LifeSteps for PrEP for Youth
MC: Management Core
mHealth: mobile health
NIH: National Institutes of Health
PI: principal investigator
PrEP: pre-exposure prophylaxis
RCT: randomized controlled trial
SMS: short message service
SRV: subject recruitment venue
STI: sexually transmitted infection
TC: Technology Core
UNC: University of North Carolina
YAB: youth advisory board
YMSM: young men who have sex with men
